# The Effect of Banana Fiber and Banana Peel Fiber on the Chemical and Rheological Properties of Symbiotic Yogurt Made from Camel Milk

**DOI:** 10.1155/2021/5230882

**Published:** 2021-12-15

**Authors:** Younes Safdari, Mohsen Vazifedoost, Zohreh Didar, Bahareh Hajirostamloo

**Affiliations:** Department of Food Science and Technology, Neyshabur Branch, Islamic Azad University, Neyshabur, Iran

## Abstract

Functional foods play an important role in human health by prevention of disease. A variety of functional foods are produced around the world. Recently, the consumption of dairy products containing probiotic bacteria and prebiotics (synbiotic) has increased. Yoghurt is the most common fermented dairy product. Various compounds are used to enrich yoghurt. One of these compounds is dietary fiber. Since the peel of fruits has a significant amount of fiber and is mainly disposed of as solid waste, so using the peel of fruits to extract fiber can not only solve environmental problems but also produce a cheap and useful source that leads to the production of dietary fiber. In this study, the effect of banana fiber and banana peel fiber at different concentrations (0, 0.2, 0.5, and 1%) on the chemical and rheological properties of synbiotic yogurt prepared from camel milk was investigated. The result showed that with increase of the amount of both fibers, pH, hydration, surface tension, overall acceptability, color, and flavor of the samples decreased significantly, but the viscosity, survival of probiotic bacteria (*Lactobacillus casei* and *Lactobacillus gasseri*), and texture acceptance increased significantly (*p* < 0.05). In conclusion, these fibers were able to reduce the syneresis of yogurt, which is one of the biggest disadvantages of yogurt, and help to increase health.

## 1. Introduction

In recent years, the consumption of functional products that protect humans against stress and various diseases has increased. Dairy products, especially yogurt, due to their rich composition of protein, essential fatty acids, and minerals such as calcium and phosphorus are considered as important human diet. One of the methods of producing functional foods is the use of probiotics which include *Lactobacillus* and *Bifidobacterium* in food products [1, 2]. Probiotics have a great effect on the body's function, which can reduce blood cholesterol and prevent intestinal diseases, is an anticancer, and can improve the function of the gastrointestinal tract and immune system. For probiotics to be effective, their number must be at least 10^6^ to 10^8^ CFU [3].

Synbiotic yogurt contains both probiotic and prebiotic agents simultaneously [4]. Prebiotics increases the number of probiotics by stimulating growth or increasing metabolic activity. For example, adding inulin to fermented milk maintains the population of *bifidobacteria* during the shelf life of the product [5].

Fibers are among the “prebiotic compounds” that are mainly supplied from the cell wall of fruits, vegetables, and grains and include polysaccharides, oligosaccharides, and lignin. Banana peel makes up about 40% of all fresh fruit, which is considered a good source of food antioxidants against cancer and heart disease, but it is disposed of as waste at a high cost [6, 7].

Due to the positive effects of banana peel fiber, its use can lead to higher consumption of products such as yogurt, which can help with a lack of fiber in the diet. Dairy products can be a suitable carrier for fiber. Some researchers have been reporting the use of fiber in dairy products. According to researches, increasing fiber reduces the sensory properties of the final product [8].

Extensive studies have been conducted on the milk of animals such as buffalo, sheep, goat, and camel and its nutritional effects [9–11]. Camel milk has been considered as a rich source of protein (2.5-9.2%) and unsaturated fatty acids (43%). Camel milk compared to cow's milk has 5 times potassium and vitamin C, 4 times more sodium, 3 times more calcium and magnesium, and more unsaturated fatty acids, chlorine, folic acid, and lactoferrin protein [12, 13]. Camel milk is lower in fat and lactose than cow's milk and contains insulin-like substances, so it is a good choice for diabetics [14].

Camel milk has been suggested as an alternative for children who are allergic to cow's milk. Camel milk proteins also lack alpha-S casein and beta-lactoglobulin, while cow's milk contains a high percentage of both compounds. Camel milk has anticancer, antitumor, antiallergic, antidiabetic, antiviral, and antibacterial effects due to compounds such as lysozyme, lactoferrin, and insulin-like proteins that are resistant to gastric acidity and lactoperoxidase. Camel milk reduces oxidative stress and partially cures autism as well as other neurological diseases by increasing serum levels of glutathione, superoxide dismutase, and myeloperoxidase. In addition, it reduces cholesterol, due to the presence of protein-derived peptides [15].

Therefore, due to the biological compounds in camel milk and its beneficial effects on health, people are advised to consume milk and its fermented products. The purpose of the present study was the investigation the effect of banana fiber and banana peel fiber at different concentrations (0, 0.2, 0.5, and 1%) on chemical and rheological properties of synbiotic yogurt prepared from camel milk.

## 2. Material and Methods

### 2.1. Material

The camel milk was purchased from the Faculty of Veterinary Medicine (Ferdowsi University of Mashhad, Iran). The starter powder of DVS (Chr Hansen, ABY-10, Denmark), containing *Lactobacillus delbrueckii* subspecies *Lactobacillus bulgaricus* and *Streptococcus thermophilus*, and commercial probiotic starter (*Lactobacillus casei* and *Lactobacillus gasseri*) were purchased from the Kristin Hansen Company (Denmark). All chemicals were purchased from Merck (Germany).

### 2.2. Banana Fiber Extraction

Banana fruit was prepared from the local market (Neyshabur province, Iran). After peeling, it is cut into smaller pieces and dried in an oven (Parsion Teb, Iran) at 103°C. Then, it was ground using a grinder, and powder was kept in polyethylene containers at 25°C.

To extract fiber, 3 g of dried powder sample mixed with distilled water in a hot water bath at 60, 75, and 90°C was stirred and heated by a magnetic stirrer (Topolino, IKA, Germany) for 50, 100, and 150 minutes. Next, the samples were filtered twice, and a centrifuge (Hermle Labortechnik GMBA, Z206A, Germany) was used at 4000 rpm for 20 minutes to separate the suspended particles. After centrifugation, to separate the solid particles from the liquid supernatant extract, it was passed through filter paper no. 41. The fiber-containing extract was placed in the refrigerator and mixed with 96% ethanol with a ratio of 1 : 2. The fiber was separated from the liquid by centrifugation at 4000 rpm for 15 minutes. The supernatant was removed, and the remaining precipitate was washed with 70% ethanol and then with 96% ethanol to remove impurities. Finally, the samples were dried by freeze-drying and packaged in polyethylene bags and stored in the refrigerator [16].

### 2.3. Fiber Properties

#### 2.3.1. Swelling

To calculate swelling, 0.2 g of the sample in a 10 ml container was mixed with distilled water. After 12 hours, the swollen sample volume was read and the swelling was expressed in ml/g [17].

#### 2.3.2. Water Holding Capacity (WHC)

1-2 g of dietary fiber was weighed and transferred to a centrifuge tube. 30 ml of distilled water was added to the contents of the centrifuge tube and allowed to stand for one hour. Then, it was transferred to a centrifuge of 2000 g for 15 minutes. The contents of the centrifuge tube were then carefully weighed, and WHC was measured using following formula [18]:

(1) $$\begin{array}{c} \text{WHC}=\frac{\text{wet weight}-\text{dry weight}}{\text{dry weight}}. \end{array}$$

### 2.4. Camel Milk Characteristics

Total dry matter was measured by oven at 105°C. The protein content was measured using the Kjeldahl method according to AOAC methods. Ash measurement was performed using the AOAC method [19]. The fat content of the samples was determined by the Gerber volumetric method using a butyrometer [20].

### 2.5. Preparing Synbiotic Yogurt

To produce synbiotic yogurt made from camel milk, banana fiber and peel banana fiber were added to camel milk in 4 concentrations (0, 0.2, 0.5, and 1%). After homogenization of the mixture by UltraTrax at 9000 rpm, the thermal process was performed at 95° C by steam bath for 5 minutes. Subsequently, the milk temperature was reduced to the inoculation temperature (45°C). Then, 0.1% of DVS powder containing a mixture of yogurt starter bacteria and probiotics (*Lactobacillus casei* and *Lactobacillus Gasseri*) was added. Afterward, the samples were kept in the incubator at 42°C for fermentation until the pH of the yogurt reached 4.5-6.6. The samples were then transferred to the refrigerator at 4°C and stored until all tests were performed [21].

### 2.6. Synbiotic Yogurt Properties

#### 2.6.1. Syneresis

About 30 g of the sample was weighed into a 50 ml falcon, and then, the tubes were centrifuged at 2500 rpm for 10 minutes. After centrifugation, the released serum was weighed and the syneresis was calculated from the following equation [21]:

(2) $$\begin{array}{c} \text{Syneresis}=\left.\text{weight of the whey after filtration}/\text{weight of the yogurt sample}\right)\times 100. \end{array}$$

#### 2.6.2. pH

The pH of the samples was measured using a digital pH meter (Metrohm 827, Switzerland) at 25°C. The pH meter was calibrated using buffer solutions of pH 7 and 4 [21].

#### 2.6.3. Rheological Properties

The viscosity of the samples was measured using a rotation viscometer (Brookfield DV-III Ultra, model RV, USA) at 25°C and shear rate range of 0 to 85 S^−1^ [21].

#### 2.6.4. Interfacial Tension

The interfacial tension of the samples was measured using the ring method by a tensiometer (KRUSS, MK 100, Germany) equipped with an electric temperature regulation system at 25°C.

#### 2.6.5. Sensory Evaluation

The organoleptic properties of synbiotic yogurt samples (i.e., color, aroma, texture, taste, and overall acceptability) were evaluated using the hedonic method by 6 panelists (1 male and 5 female) in three replications.

#### 2.6.6. Survival of Probiotic Bacteria in Yogurt

MRS agar medium (Mirmidia, Iran) containing 0.05% of bile salt was used to count the probiotic bacteria. The peptone water solution was also used for dilution. To prepare a dilution of 0.1, 1 g of sample was mixed with 9 ml of Peptone Water solution and mixed completely. Similarly, subsequent dilutions were prepared by transferring 1 ml of each dilution to 9 ml of peptone water solution. *Lactobacillus casei* and *Lactobacillus gasseri* were counted. The plates were incubated under aerobic and anaerobic conditions at 37°C for 72 hours. Anaerobic conditions were created using an anaerobic jar [21].

### 2.7. Statistical Analysis

The results were analyzed in a factorial design based on a completely randomized design using SPSS software (version 24). The mean values were analyzed using Duncan's test at 5% (*p* < 0.05).

## 3. Results and Discussion

### 3.1. Chemical Compounds of Camel Milk

The main chemical constituents of the camel milk sample contained 4.27% fat, 3.37% protein, 7.56% dry matter, and pH of 6.34, which was according to the values reported in other studies [22].

### 3.2. Physical Properties of Banana Fiber and Banana Peel Fiber

The rate of swelling of banana fiber and its peel was approximately 5.8 ml/g. These results were similar to the swelling of wheat and pear fiber. Figuerola et al. [23] reported a range of 6.8-1.6 for grape fiber, apples, lemons, and oranges.

The water holding capacity (WHC) of banana fiber was significantly higher than banana peel fiber (2.80 and 2.66, respectively), which could be due to the presence of higher amounts of soluble dietary fiber and protein in the banana fiber. Soluble dietary fiber and protein have hydrophilic groups that increase water absorption.

### 3.3. The Physicochemical Properties of Synbiotic Yoghurt

#### 3.3.1. pH

Table 1 shows the effect of different amounts of banana fiber on pH changes in yogurt samples. As can be seen, with increase of the banana fiber and banana peel fiber, the pH of yogurt samples decreased significantly (*p* < 0.001).

The results showed that in all samples, the pH decreased compared to the control sample. With the increase of banana fiber and banana peel fiber, the pH decreased. The lowest pH of the sample was related to the 1% fiber and the highest pH was related to the control sample. The reason for the decreasing pH with increasing fiber may be related to the prebiotic effect of fiber on improving the activity and growth of probiotic bacteria, which has been previously reported [21]. In other words, by increasing the activity of probiotic bacteria, the production of acidic metabolites resulting from their activity has increased, which can play a role in reducing the final pH of the product.

Another reason for the decrease in pH is increasing the dry matter of the product with the addition of fiber. This can increase the acidity and decrease the pH.

Salwa et al. [24] reported that yogurt containing carrot extract has a lower pH than control yogurt due to its organic acids as well as sugar fermentation and acid production due to bacterial activity. Also, the results of this study were consistent with the results of Bonczar et al. [25], Tseng and Zhao [26], and Temiz et al. [27].

#### 3.3.2. Syneresis

Table 2 shows the effect of different amounts of banana fiber on the syneresis of yogurt samples. As can be seen, the increase in banana fiber and banana peel fiber significantly reduced the synthesis of synbiotic yogurt samples (*p* < 0.001). In both types of fiber, synbiotic yogurt samples with 1% fiber had the lowest syneresis rate (21.52%), and the control sample had the highest rate (30.74%).

Staffolo et al. [28] concluded that yogurt containing apple fiber and orange fiber had less syneresis. The ability of fibers to bind to water molecules and interfere with milk components, especially proteins, and thus the stability of the protein network can prevent the free movement of water and lead to a reduction in syneresis. Żbikowska et al. [29] showed that the use of inulin reduces the syneresis of yogurt and other fermented milks. Another reason for reducing the syneresis of yogurt containing prebiotics is increasing the consistency and also the capacity index of the complex with water, which allows prebiotic compounds to prevent syneresis by increasing the binding water.

Ramirez-Santiago et al. [30] also showed that enriched yogurt with potato fiber had less syneresis. Increasing the dry matter stabilizes the gel network and also increases the water binding capacity and reduces syneresis [31].

In this study, the water holding capacity of yogurt samples is more affected by the hydrocolloids. Increasing the amount of hydrocolloid increases the amount of water trapped in the gel network. However, with a further increase in the percentage of hydrocolloids, the gel structure is destroyed and its water holding capacity decreases [32].

Also, Gustaw et al. [21] used prebiotics including fructooligosaccharide, inulin, and resistant starch in yogurt and concluded that yogurt samples containing fructooligosaccharide and inulin had lower viscosity, lower firmness, and comparatively lower syneresis.

#### 3.3.3. Rheological Properties

Table 3 shows the effect of different amounts of banana fiber on the viscosity of yogurt samples. As can be seen, the increase in banana fiber and banana peel fiber significantly increased the apparent viscosity of synbiotic yogurt samples (*p* < 0.001). The highest viscosity was related to the 1% banana fiber samples, and the lowest viscosity was related to the control sample.

The fiber in yogurt samples changes its structure, strengthens the gel structure, and improves the rheological behaviors by binding to water molecules [33]. These results are in agreement with the results of other research. Increasing the water binding capacity and creating a stronger gel increases the resistance to flow. Increasing the total solid can also increase the viscosity [21].

The fiber increases the viscosity and firmness of the yoghurt. The increase in viscosity of low-fat yogurt containing soy flour may be related to the interaction between fiber, oligo- or polysaccharides, and low-fat yogurt proteins [3].

#### 3.3.4. Interfacial Tension

Table 4 shows the effect of different amounts of banana fiber on the surface tension of yogurt samples. As can be seen, the increase in banana fiber and banana peel fiber significantly reduced the surface tension of synbiotic yogurt samples (*p* < 0.001).

However, no significant difference was observed between the control sample and 0.2% banana fiber (*p* > 0.05), but there was a significant difference between the other samples (*p* < 0.05). The highest and lowest surface tensions were related to the control sample and 1% banana fiber sample, respectively.

#### 3.3.5. Sensory Evaluation

Table 5 shows the effect of banana fiber on the sensory properties of synbiotic yogurt samples.

As can be seen, with increasing fiber content, the color of yogurt samples significantly decreased compared to that of the control sample (*p* < 0.001). The highest color was related to the control sample, and the lowest was related to the 1% banana fiber sample. Also, no significant difference was observed between the control treatments and 0.2% banana fiber. As the amount of fiber increases, the amount of syneresis decreases, and due to the change in light reflection, the opacity of the product also increases.

Increasing the banana fiber caused a significant increase in the consistency of yogurt samples (*p* < 0.001).

Overall acceptance of the product showed a significant decrease with increasing amount of banana fiber (*p* < 0.001). The lowest and the highest overall acceptability were obtained for the sample containing 1% fiber and the control treatment, respectively.

Staffolo et al. [28] showed that an increase of 1.3% in fiber had no effect on sensory properties, but increasing this value caused a significant sensory decrease. Sendra et al. [33] also showed that the increase in fiber caused a significant reduction in sensory properties, which is consistent with the results of this study.

Some studies have shown that prebiotics in the probiotic yoghurt cause improvement of the texture of the product; however, over time, as the production of acid increases and the pH decreases, the sensory properties of the final product decrease. Similar findings have been reported previously [34–36].

#### 3.3.6. Survival of Probiotic Bacteria in Yogurt

Table 6 shows the effect of different amounts of banana fiber on the population of *Lactobacillus casei* and *Lactobacillus gasseri* in yogurt samples. As can be seen, with increasing banana fiber and banana peel fiber, the survival of *Lactobacillus casei* and *Lactobacillus gasseri* increased significantly (*p* < 0.01).

As can be seen, the highest survival of *Lactobacillus casei* and *Lactobacillus gasseri* was for the sample of 1% banana fiber and the lowest was for the control sample. The survival of *Lactobacillus casei* and *Lactobacillus gasseri* can be attributed to the prebiotic compounds of banana fiber and its peel. Prebiotic compounds increase the growth and activity of probiotics.

Sendra et al. [33] claimed that the survival of probiotics due to the increase in fiber, the rapid conversion of lactose to lactic acid, and the interaction of the milk components (mainly proteins) stabilize the protein and prevent the transfer of free water.

Another reason for the increase in probiotics by increasing the amount of banana fiber is to increase the buffering capacity of the fibers, which protects the bacteria against stressful conditions [32]. The increase in survival of bacteria is also due to the increase of the food required by probiotic bacteria to provide nitrogen and carbon sources and suitable environmental conditions for further growth and activity of this microorganism.

## 4. Conclusion

Functional yogurt contains fiber has health effects on humans. In this study, the effects of the banana fiber and banana peel fiber on physicochemical, rheological, and sensory properties of synbiotic yogurt using camel milk and probiotic bacteria (*Lactobacillus casei* and *Lactobacillus gasseri*) were investigated. The banana fiber and banana peel fiber increased viscosity, the survival of probiotic bacteria, and consistency but decreased pH, syneresis, surface tension, and sensory properties (color, flavor, and overall acceptability). In all samples, the effect of banana fiber was much greater than the peel fiber. It can be concluded that these two types of fiber can have healing properties for the body.

## Figures and Tables

**Table 1:** The effect of banana fiber on pH changes in yogurt samples.

Treatment	Concentrations (%)			
	0	0.2	0.5	1
Banana fiber	4.37 ± 0.01^bB^	4.38 ± 0.01^abB^	4.41 ± 0.01^aA^	4.40 ± 0.01^aA^
Banana peel fiber	4.42 ± 0.01^aA^	4.40 ± 0.01^abA^	4.39 ± 0.01^bcB^	4.37 ± 0.01^cB^

The mean ± SD (standard deviation) within columns with different capital letters and rows with different small letters differs significantly (*p* < 0.05).

**Table 2:** The effect of banana fiber on syneresis changes in yogurt samples.

Treatment	Concentrations (%)			
	0	0.2	0.5	1
Banana fiber	29.16 ± 1.04^aA^	28.00 ± 1.00^abA^	26.33 ± 1.15^beA^	24.00 ± 1.001^cA^
Banana peel fiber	28.20 ± 0.70^aA^	27.00 ± 0.50^abA^	26.00 ± 0.60^bcA^	25.10 ± 0.80^cA^

The mean ± SD (standard deviation) within columns with different capital letters and rows with different small letters differs significantly (*p* < 0.05).

**Table 3:** The effect of banana fiber on viscosity of yogurt samples.

Treatment	Concentrations (%)			
	0	0.2	0.5	1
Banana fiber	2650 ± 250^cA^	3100 ± 100^bA^	3250 ± 50^abA^	3516 ± 175^aA^
Banana peel fiber	3000 ± 300^bA^	2800 ± 100^bB^	3150 ± 150^bA^	3600 ± 200^aA^

The mean ± SD (standard deviation) within columns with different capital letters and rows with different small letters differs significantly (*p* < 0.05).

**Table 4:** The effect of banana fiber on interfacial tension of yogurt samples.

Treatment	Concentrations (%)			
	0	0.2	0.5	1
Banana fiber	33.60 ± 1.5^aA^	33.43 ± 055^aA^	32.90 ± 0.50^aA^	31.50 ± 0.50^bA^
Banana peel fiber	35.16 ± 1.76^aA^	34.16 ± 0.90^aA^	31.33 ± 0.86^bA^	29.33 ± 0.92^bB^

The mean ± SD (standard deviation) within columns with different capital letters and rows with different small letters differs significantly (*p* < 0.05).

**Table 5:** The effect of banana fiber on sensory properties of yogurt samples.

Sensory characteristic	Treatment	Concentrations (%)			
		0	0.2	0.5	1
Flavor	Banana fiber	4.5 ± 0.55^aA^	4.70 ± 0.20^abA^	4.80 ± 0.20^aA^	3.20 ± 0.50^aB^
	Banana peel fiber	4.5 ± 0.50^aA^	4.20 ± 0.20^abB^	4.00 ± 0.20^abB^	3.40 ± 0.30^bA^
Texture	Banana fiber	4.20 ± 0.10^aA^	4.30 ± 0.10^aA^	4.45 ± 0.15^aA^	4.51 ± 0.10^bA^
	Banana peel fiber	3.93 ± 0.25^bA^	4.10 ± 0.30^abA^	4.50 ± 0.40^abA^	4.86 ± 0.40^aA^
Color	Banana fiber	4.10 ± 0.50^aA^	3.80 ± 0.30^aA^	3.60 ± 0.10^aA^	3.23 ± 0.25^bA^
	Banana peel fiber	4.5 ± 0.50^aA^	3.90 ± 0.30^bA^	3.5 ± 0.20^bA^	3.90 ± 0.10^bA^
Overall acceptability	Banana fiber	4.00 ± 0.30^aA^	4.03 ± 0.11^aA^	3.93 ± 0.15^aA^	3.36 ± 0.15^bA^
	Banana peel fiber	4.50 ± 0.30^abA^	4.10 ± 0.10^abA^	3.60 ± 0.30^bcA^	3.20 ± 0.20^cA^

The mean ± SD (standard deviation) within columns with different capital letters and rows with different small letters differs significantly (*p* < 0.05).

**Table 6:** The effect of banana fiber on population of *Lactobacillus casei* and *Lactobacillus gasseri* in yogurt samples.

MO	Treatment	Concentrations (%)			
		0	0.2	0.5	1
*Lactobacillus casei*	Banana fiber	7.23 ± 0.35^aA^	7.70 ± 0.20^abA^	7.80 ± 0.20^aA^	8.20 ± 0.50^aA^
	Banana peel fiber	7.00 ± 0.50^bA^	7.5 ± 0.50^abA^	8.00 ± 0.20^abA^	8.20 ± 0.30^aA^
*Lactobacillus gasseri*	Banana fiber	7.00 ± 0.40^bA^	7.35 ± 0.15^abA^	7.70 ± 0.30^aA^	7.80 ± 0.20^aA^
	Banana peel fiber	6.50 ± 0.50^bA^	6.76 ± 0.25^abA^	7.00 ± 0.50^abA^	7.80 ± 0.30^aA^

The mean ± SD (standard deviation) within columns with different capital letters and rows with different small letters differs significantly (*p* < 0.05).

## Data Availability

The data used to support the findings of this study are available from the corresponding author upon request.
